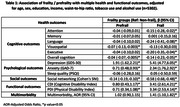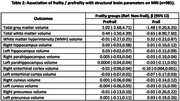# Impact of Physical Frailty on Cognitive, Psychological, Social, Functional Outcomes, Multimorbidity and Brain Structure among Aging Rural Indians

**DOI:** 10.1002/alz.092660

**Published:** 2025-01-09

**Authors:** Jonas S Sundarakumar, Pravin Sahadevan, Raghav Prasad, Pooja Rai, Hitesh Pradhan, Sakshi Arora, Sumedha Mitra, Priya Chatterjee, Aishwarya B Hiremath, Palash K Malo, Ramya Burra, CBR‐SANSCOG Study Team

**Affiliations:** ^1^ Centre for Brain Research, Indian Institute of Science, Bangalore, Karnataka India

## Abstract

**Background:**

Though frailty is common in aging, its impact on varied health outcomes has been grossly understudied among rural Indians. We aimed to cross‐sectionally examine the impact of physical frailty on 13 health outcomes and brain structure in this population.

**Method:**

Participants (n=5302) were non‐demented, aging individuals (≥45 years) from the ongoing Srinivaspura Aging Neuro Senescence and Cognition (SANSCOG) cohort in rural southern India. Physical frailty was evaluated using a modified version of Fried's criteria, and participants were classified as non‐frail, pre‐frail and frail. Cognitive functioning was assessed using a comprehensive, culture‐ and education‐fair battery (COGNITO) spanning multiple cognitive domains. Psychological assessments included Generalized Depression Scale (GDS‐30), Generalized Anxiety Disorder (GAD‐7) scale and Pittsburgh Sleep Quality Index (PSQI). Cohen’s Social Networking Index (SNI) and Instrumental Activities of Daily Living for Elderly (IADL‐E) were used to assess social networking and functional outcomes, respectively. Multimorbidity was assessed using self‐reported/objective measures. A subset of participants (n=981) underwent 3‐Tesla brain MRI. Regional brain volumes (total grey matter, total white matter, right/left hippocampus, parahippocampus, entorhinal cortex, cuneus and precuneus) and WMH volume were derived using standard protocols. Multivariable Linear/logistic regression models were used, adjusting for age, sex, education, income, waist‐to‐hip ratio, tobacco and alcohol use.

**Result:**

Frailty was significantly associated with poorer cognitive performance, overall (β=‐0.14 [‐0.23,‐0.05] and for attention (β=‐0.15 [‐0.28,‐0.02]), language (β=‐0.24 [‐0.41,‐0.08], visuospatial (β=‐0.13 [‐0.30,0.03]) and executive functioning (β=‐0.20 [‐0.35,‐0.04]), higher depression (β=‐5.41 [4.77,6.05]) and anxiety (β=‐2.34 [1.76,2.92]) scores, poorer sleep quality (β=0.05 [‐0.50,0.59]), lower SNI scores (β=‐0.58 [‐0.68,‐0.48]), higher cognitive (β=3.41 [2.61,4.21]) and physical disability (β=3.34 [2.53,4.14]) scores and higher odds of multimorbidity (AOR=1.41 [1.10,1.82]). Prefrailty was associated with poorer overall cognition (β=‐0.04 [‐0.07,‐0.005]) and visuospatial ability (β=‐0.07 [‐0.13,‐0.003], higher depression (β=1.91 [1.62,2.21]) and anxiety (β=0.71 [0.48,0.93]) scores, lower SNI scores (β=‐0.14 [‐0.19,‐0.10]), higher cognitive (β=0.80 [0.43,1.17]) and physical disability (β=0.71 [0.34,1.08]) scores. Frailty and prefrailty were not significantly associated with any of the brain MRI parameters.

**Conclusion:**

Physical frailty is associated with multiple adverse health outcomes but not with brain structural parameters, cross‐sectionally. Early identification and mitigation of frailty could potentially reduce dementia risk and improve overall health and functioning in the aging population.